# Skin and bone: a mysterious link to an underdiagnosed cause of osteonecrosis

**DOI:** 10.1016/j.ero.2025.02.002

**Published:** 2025-03-27

**Authors:** Pierre-Marie Duret, Simon Gravier, Xavier Parent, Laurent Messer, Antoine Mahé

**Affiliations:** 1 Rheumatology Department, Hôpitaux Civils de Colmar, Colmar, France; 2 Department of Infectious and Tropical Diseases, Hôpitaux Civils de Colmar, Colmar, France; 3 Laboratory Biochemistry, Hôpitaux Civils de Colmar, Colmar, France; 4 Dermatology Department, Hôpitaux Civils de Colmar, Colmar, France

A 45-year-old woman from Chad with HIV infection (undetectable viral load and 2258 CD4+ T cells/μL [N: 700-1100 T cells/μL]) receiving highly active antiretroviral therapy with bictegravir, emtricitabine, and tenofovir alafenamide presented to our rheumatology clinic with knee pain lasting for 1 year. Radiographs showed bilateral extensive epiphyseal-metaphyseal bone infarctions of tibial plateaus and femoral condyles (Fig, A), confirmed by magnetic resonance imaging (MRI) (Fig, B). A diagnosis of bilateral knee osteonecrosis (ON) was made. Laboratory workup ruled out major aetiologies of ON, such as hypertriglyceridemia, antiphospholipid syndrome, and sickle cell disease. Low serum levels of adrenocorticotropic hormone (ACTH <0.7 pmol/L; [N: 1.6-13.9 pmol/L]) and cortisol (6 nmol/L; [N: 166-507 nmol/L]) were observed, along with low urine free cortisol (UFC) levels (<3 nmol/L; [N: 16-170 nmol/24 hours]).

**Figure:**
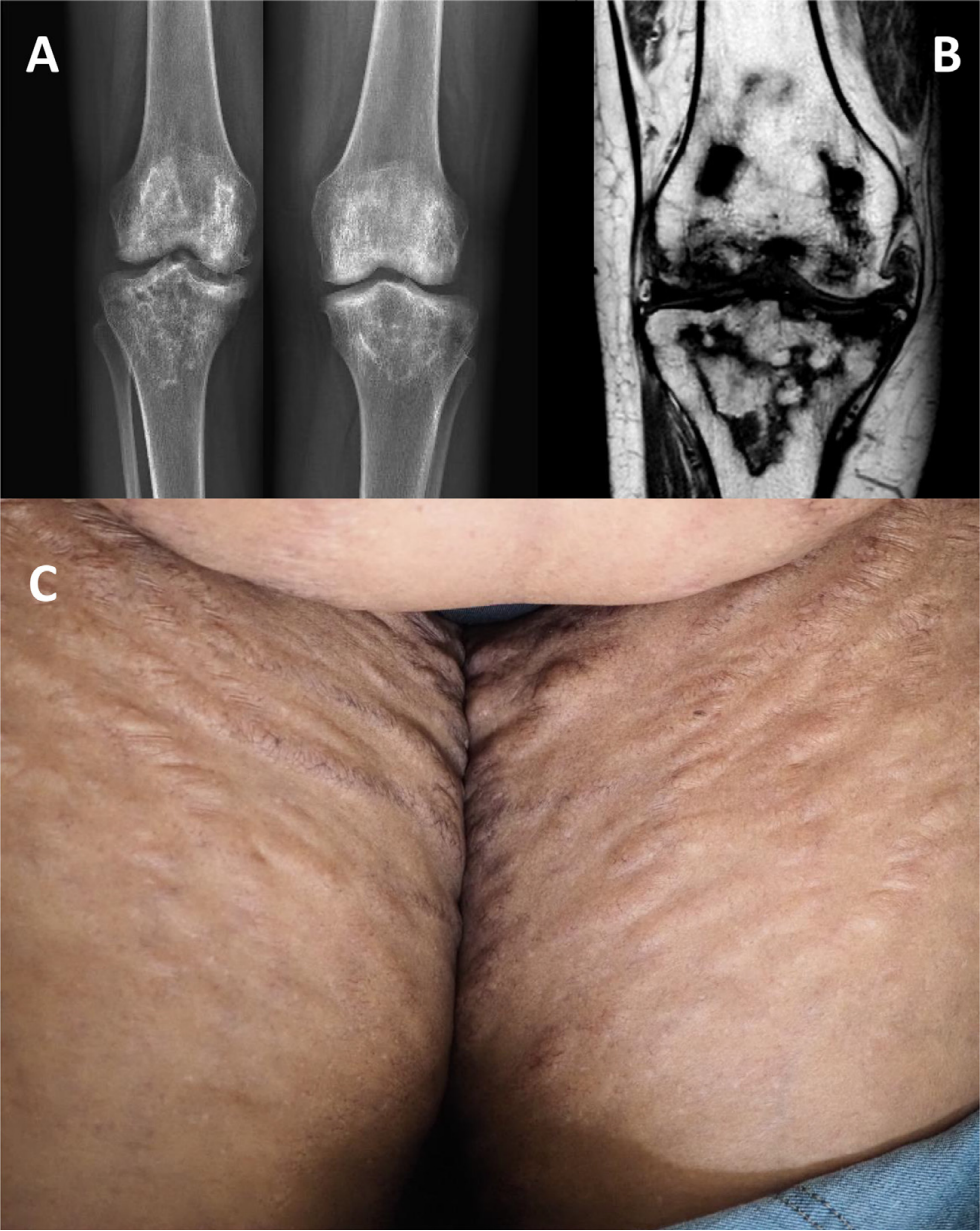
(A) Radiographs showing bilateral knee osteonecrosis. (B) Magnetic resonance imaging (MRI) (T1-weighted spin echo) of the right knee showing epiphyseal-metaphyseal femoral and tibial infarction lesions. (C) Global cutaneous lightening and diffuse large atrophic stretch marks on the thighs induced by chronic misuse of corticosteroid-containing cosmetics for skin bleaching.

The biological features of altered cortisol metabolism were characteristic of exogenous corticosteroid administration, a classic inducer of ON.

In 2014, we reported the case of a 26-year-old man from the Democratic Republic of Congo who presented with osteonecrosis of the femoral head associated with suppression of the hypothalamic–pituitary–adrenal axis. This was linked to the use of massive quantities of clobetasol propionate–based creams for cosmetic skin bleaching [1]. In the present case, although the patient did not acknowledge any hidden drug misuse, skin examination revealed global cutaneous lightening, diffuse large atrophic stretch marks on the thighs (Fig C), and a poikilodermic appearance of the neck, features strongly suggestive of prolonged use of corticosteroids. Therefore, ON related to the use of corticosteroid-based topical creams for skin bleaching purposes was suspected. Liquid chromatography–mass spectrometry testing a panel of corticosteroids in peripheral blood revealed high concentrations of betamethasone (16.9 ng/mL) [2]. The patient finally conceded chronic use of corticosteroid-containing cosmetics. In the previous case, extensive use of a specific clobetasol propionate cream was established, whereas in the present one, betamethasone was detected in the serum, a compound known to be present in cosmetics used during skin bleaching practices [3]. Both patients were natives of Sub-Saharan Africa, an area of the globe where cosmetic skin bleaching is common [4], and both patients acknowledged long-term use of such products.

The identification of the only 2 reported cases to our knowledge of this condition within the same centre, while serving a population with relatively low ethnic diversity, suggests the following:

• Skin bleaching–related ON is probably more prevalent than these 2 cases would indicate and therefore might be underdiagnosed worldwide due to clinicians’ lack of awareness about this practice.

Underrecognition of skin bleaching practices involving the misuse of hidden corticosteroid-containing cosmetics and the subsequent underdiagnosis of its related systemic complications, including ON, should be considered detrimental to communities already experiencing health disparities.

Therefore, we recommend considering the misuse of corticosteroid-based cosmetic products for skin bleaching as a potential cause of ON, particularly in individuals from communities where this practice is prevalent.

## Funding

This research did not receive any specific grant from funding agencies in the public, commercial, or not-for-profit sectors.

## CRediT authorship contribution statement

**Pierre-Marie Duret:** Conceptualization, Investigation, Methodology, Supervision, Writing – original draft, Writing – review & editing. **Simon Gravier:** Investigation, Writing – review & editing. **Xavier Parent:** Investigation, Writing – review & editing. **Laurent Messer:** Investigation, Writing – review & editing. **Antoine Mahé:** Conceptualization, Investigation, Methodology, Writing – original draft, Writing – review & editing.

## Competing interests

All authors declare they have no competing interests.
